# Impact of cigarette smoke and aerobic physical training on histological and molecular markers of prostate health in rats

**DOI:** 10.1590/1414-431X20209108

**Published:** 2020-04-17

**Authors:** A.S.C. Veras, D.B. Baptista, N.J. dos Santos, H.H.A. Thorpe, P.M. Seraphim, A.R. Florido, G.R. Teixeira

**Affiliations:** 1 Programa de Pós-Graduação em Ciências da Motricidade, Universidade Estadual Paulista, Presidente Prudente, SP, Brasil; 2 Departamento de Educação Física, Universidade Estadual Paulista, Faculdade de Ciências e Tecnologia, UNESP, Presidente Prudente, SP, Brasil; 3 Programa de Pós-Graduação em Biologia Celular e Estrutural, Universidade de Campinas, Campinas, SP, Brasil; 4 Department of Biomedical Sciences, Ontario Veterinary College, University of Guelph, Guelph, ON, Canada; 5 Departamento de Fisioterapia, Faculdade de Ciências e Tecnologias, Universidade Estadual Paulista, Presidente Prudente, SP, Brasil; 6 Programa de Pós-Graduação em Fisiologia, Universidade de São Paulo, São Paulo, SP, Brasil

**Keywords:** Inflammation, Androgen receptor, Growth factors, Endurance exercise, BAX, BCL-2

## Abstract

Recent evidence suggests that aerobic physical training may attenuate the deleterious effects of cancer risk factors, including smoking. We investigated the effects of cigarette smoke inhalation and aerobic physical training on the expression of steroid receptors and inflammatory and apoptotic proteins in the prostate. Forty male Wistar rats were distributed in four groups: control (CO), exercise (EXE), cigarette smoke exposure (CS), and cigarette smoke exposure with exercise (CS+EXE). For eight weeks, animals were repeatedly exposed to cigarette smoke for 30 min or performed aerobic physical training either with or without the cigarette smoke inhalation protocol. Following these experiments, we analyzed prostate epithelial morphology and prostatic expression of androgen (AR) and glucocorticoid receptors (GR), insulin-like growth factor (IGF-1), B-cell lymphoma-2 (BCL-2), BCL-2-associated X protein (BAX), interleukin-6 (IL-6), tumor necrosis factor-alpha (TNF-α), and nuclear factor-kappa B (NF-κB) via immunohistochemistry. Cigarette smoke exposure stimulated the expression of AR, IGF-1, BCL-2, and NF-κB while downregulating BAX, IL-6, and TNF-α labeling in the prostate. In contrast, aerobic physical training attenuated cigarette smoke-induced changes in AR, GR, IGF-1, BCL-2, IL-6, TNF-α, and NF-κB. This suggests that cigarette smoke stimulates inflammation and reduces apoptosis, culminating in increased prostatic epithelial and extracellular matrices, whereas physical training promoted beneficial effects towards maintaining normal prostate morphology and protein levels.

## Introduction

Cigarette smoking is considered one of the greatest risk factors in the development of cardiovascular disease and is a potent metabolic stimulator in the progression of various types of cancer, including prostate cancer (1). Studies indicate that secondhand or sidestream smoke contains higher concentrations of toxic components than mainstream smoke, and secondhand smoke exposure is a factor in the development of smoking-related diseases (2). The toxic components of cigarette smoke alter the mitogenic and anti-apoptotic properties of hormonal peptides, resulting in changes in metabolism through dysregulation of proliferation, epithelial differentiation, and apoptotic inhibition (3).

Current data provide convincing evidence that physical exercise reduces the risk of lung, prostate, and endometrial diseases (4). Although the most optimal intensity, volume, and modality of exercise to combat disease have yet to be established in the literature, there are reports that moderate physical exercise increases apoptosis in cancer cells (5). Tobacco-induced alterations to prostate tissue proliferation and chronic inflammation have been reported in clinical populations (6). While it is possible that exercise promotes beneficial reductions in prostate cell proliferation and inflammation caused by smoke exposure, there are currently no preclinical studies evaluating the interaction between exercise, prostatic health, and smoking. Notably, physical exercise can modulate cellular activity in the rat prostate by reducing androgen receptor (AR) activation, which in turn reduces cellular proliferation (5). Glucocorticoid receptor (GR) activation is also implicated in prostatic health and exercise, as glucocorticoid administration in rodents conduces hormonal regulation in the prostate and aerobic physical training increases plasma glucocorticoid levels, consequently inducing intracellular GR activation (7). Glucocorticoids such as cortisol in humans and corticosterone in rats are steroid hormones produced by the adrenal glands and regulated by the hypothalamic-pituitary-adrenal axis. These molecules play a fundamental role in controlling physiological processes, including development, inflammation, and stress responses (8). Evidence suggests that glucocorticoids are integral to programmed cell death and the inhibition of cell proliferation in androgen-independent prostatic adenocarcinomas (7).

Although cigarette smoking is a clear factor in the development of prostate disorders, the molecular mechanisms of smoking-related carcinogenesis are complex and involve the dysregulation of many proteins and pathways. Kenfield and colleagues (9) evaluated correlations between inflammatory features and smoking in 5,366 prostate cancer patients and found that smoking and inflammation substantially increased mortality rates and cancer recurrence. Substantial increases in levels of the transcription factor complex nuclear factor kappa-light-chain-enhancer of activated B cells (NF-κB) by pro-inflammatory cytokines, including interleukin-1 (IL-1), IL-6, and tumor necrosis factor α (TNF-α), have been observed in plasma and prostate samples of tobacco users (6). Reactive oxygen species generated by cigarette smoking have been linked to an increase in inflammatory gene expression through the induction of NF-κB and proinflammatory mediators, leading to inflammation and epithelial disease progression (8). Aerobic physical training of moderate-intensity (40-60% VO_2_ max) decreases circulating levels of inflammatory cytokines IL-6 and TNF-α while concurrently increasing plasma levels of IL-10 (10). However, while cigarette smoking and aerobic physical exercise may independently alter prostate cell metabolism, the relationship between smoking and physical exercise on prostatic health is unclear. We investigated the ability of cigarette smoke inhalation and aerobic physical training, alone or in combination, to modulate inflammatory, apoptotic, and steroid receptor protein expression in the rat prostate. We found that exercise had beneficial prospects in maintaining prostate health, even in animals chronically exposed to cigarette smoke.

## Material and Methods

### Animals and experimental design

All animal protocols were approved by the Ethics Committee on the Use of Animals (CEUA) of the School of Technology and Sciences at São Paulo State University (UNESP), campus of Presidente Prudente (Protocol No. 1/2012). All procedures were conducted following the guidelines for experimentation with animals according to the Guide to the Care and Use of Laboratory Animals published by the United States National Institutes of Health.

**Figure 1:**
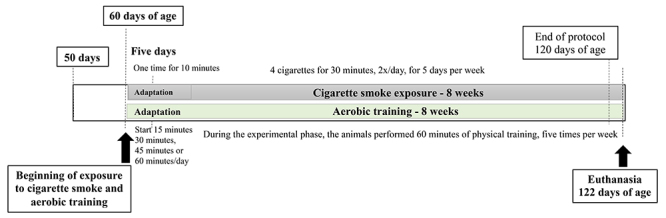
Timeline of experimental design during the aerobic training and cigarette smoke exposure.

Forty post-pubertal Wistar rats from the Central Animal Laboratory at UNESP Botucatu were maintained at an average temperature of 22±2°C under a 12-h light/dark cycle in solid-bottomed polyethylene cages (40×30×15 cm). Animals had *ad libitum* access to filtered tap water and standard food chow. Prior to testing, animals were randomly divided into one of four groups: control (CO; n=10), which remained inactive for the duration of the study; cigarette smoke inhalation (CS; n=8, as two animals died during the course of the study), which were subjected to cigarette smoke inhalation; exercise (EXE; n=10), which were subjected to an aerobic physical exercise protocol as described below; and cigarette smoke inhalation with exercise (CS+EXE; n=10), which were exposed to both smoke inhalation and the aerobic physical exercise protocol (Figure 1).

### Exposure to cigarette smoke

At 60 days of age, animals in the CS and CS+EXE groups were exposed to cigarette smoke in two phases: adaptation and experimental. In the adaptation phase (first five days of experimentation), the animals were exposed to the smoke of two cigarettes for 10 min once per day. During the experimental phase, animals were exposed to smoke from four cigarettes for 30 min twice per day, five days per week for eight weeks. At these doses, animals were exposed to 250–350 ppm of carbon monoxide (11). The animals were placed in one of two compartments in a two-sided chamber, and the cigarettes were burned separately in the other compartment. An air compressor (10 L/min) was used to ensure the passage of smoke to the animal through a hole connecting the two compartments. Commercially-available cigarettes (Malboro Red^®^) were composed of a mixture of tobacco, sugars, cigarette paper, and vegetable extracts; flavoring agents were used. As reported on the packaging, each cigarette produced 10 mg of tar, 0.8 mg of nicotine, and 10 mg of carbon monoxide when burned.

### Physical training protocol

At 60 days of age, the exercise groups performed aerobic physical training on a treadmill at 10 m/min as previously described (12). Animals underwent an adaptation phase for one week followed by an eight-week experimental phase. During the adaptation phase, the animals performed aerobic physical training on a treadmill for either 15, 30, 45, or 60 min per day. During the experimental phase, the animals performed 60 min of physical training five times per week.

### Body mass index and nutritional analyses

Body weights were measured using a Shimadzu electronic scale (model BL3200H, Japan) once per week throughout the study. Energy intake (EI; 3 kcal/g), which was assessed weekly, was used to calculate ingested energy (EI (kcal/100 g per day) = average food intake [g] per day × 100 / animal weight × 3 kcal [g/100 g per day]). Feed efficiency (FE) was calculated as FE (g/kcal) = mean weight gain / EI, as previously described (13). To evaluate weight gain, we calculated body mass gain (Δ = final weight – starting weight). Body mass index (BMI) was evaluated using the weight and nose-to-anus length (BMI: body weight [g] / length [cm^2^]) (13).

### Biochemical analyses

Blood samples were drawn from the portal vein after 12 h of fasting. The collection was done using a syringe without an anticoagulant. After collection, blood was centrifuged for 10 min at 1008 *g* and stored at −80°C. Triglyceride content, total cholesterol, and glucose serum levels were determined using colorimetry assay (Labtest, Brazil; ZenBio, Inc., USA).

### Histopathological analysis

Prostate tissue was fixed in formalin solution for 24 h, underwent a routine of paraplastic inclusion, and cut into 5-µm sections. The sections were stained with hematoxylin and eosin (HE) to analyze histological morphology in the prostate. Using a Weibel reticle of 160 points, the stereology (acini, epithelium, and stroma) of prostate cross-sections was quantified across 10 images per slide, and 10 slides were analyzed per prostate collected. Images were obtained using an AxioCam ER.c 5s (Zeiss Primo Star, Germany) photomicroscope with a 40× objective at the Department of Physical Education of São Paulo State University (UNESP).

### Immunohistochemistry protocol

Prostate sections were mounted on glass slides, dewaxed with xylene and alcoholic gradations, and washed in running water. The slides were placed in a microwave oven in sodium citrate buffer (0.01 M, pH 6.0) for 15 min. Thereafter, slides were submerged in a 3% hydrogen peroxide solution diluted in methanol for 15 min and a 3% bovine serum albumin solution for one hour for protein blockade. Sections were then incubated with AR (N-20, sc-816, Santa Cruz Biotechnology, USA, 1:100), GR (M-20, sc-1004, Santa Cruz Biotechnology, 1:100), BCL-2 (N-19, sc-492, Santa Cruz Biotechnology, 1:250), BAX (P19, sc-526, Santa Cruz Biotechnology, 1:300), IL-6 (M-19, sc-1265, Santa Cruz Biotechnology, 1:60), TNF-α (N-19, sc-1350, Santa Cruz Biotechnology, 1:80), and NF-κB (A, sc-109, p65 subunit, Santa Cruz Biotechnology, 1:100) primary antibodies in a wet chamber overnight at 4°C. After incubation, sections were washed in sodium phosphate buffer and incubated for two hours with a secondary antibody (mouse anti-goat immunoglobulin G-horseradish peroxidase IgG-HRP, sc-2354, Santa Cruz Biotechnology, 1:200; mouse anti-rabbit IgG-HRP, sc-2357, Santa Cruz Biotechnology, 1:200). The sections were then exposed to diaminobenzidine (DAB) and counterstained with hematoxylin. For all markers, positive and negative controls were performed (14). As the positive control, we used several dilutions of the antibody in the prostate tissue to adjust the protocol associated with the negative control.

The intensity of immunoreactivity to IGF-1, BCL-2, BAX, IL-6, TNF-α, and NF-κB antigens was examined in 10 fields per prostate using ImageJ software (version 1.50i, NIH, USA) by analyzing the percentage of tissue labeling in each field and the absorbance of IGF-1, BCL-2, BAX, IL-6, TNF-α, and NF-κB immunopositive cells were used for percentage for area.

For the AR and GR quantification, the labeling indexes for each group were estimated as the percentage of stained-positive epithelial cells. The average AR and GR indexes in each group were then obtained in 1,000 secretory epithelial cells per animal from 10 sections of the intermediate region of the ventral prostate.

### Data analyses

The Shapiro-Wilk test was used to verify the normality and homogeneity of the data. Statistical analyses of tissue protein expression and anthropometric parameters were performed with one-way ANOVA and complemented with Tukey's multiple comparison test between group means. Statistical analysis of BMI values was conducted using a one-way ANOVA and Fisher's exact test. Correlation coefficients were calculated using Pearson's correlation coefficient test. Results are reported as means±SD, and P<0.05 was considered statistically significant. All statistical analysis was performed using SPSS software v.25 (IBM, USA).

## Results

### Body mass index, nutritional, and biochemical parameters

In this study, we demonstrated that exposure to cigarette smoke in both the CS and CS+EXE conditions reduced weight gain compared to the CO group and that the CS group alone showed significant reductions in total ingested energy relative to all other treatment groups. However, feed efficiency was also reduced in the CS+EXE group compared with EXE despite no significant difference in final animal weight across groups. Calculations of BMI showed that EXE and CS conditions resulted in lower values than the CO group, although interestingly, no reduction in BMI was observed in the CS+EXE condition. The EXE group had higher triglyceride plasma concentrations than the CS and CO groups, and the CS and CS+EXE groups had higher total cholesterol levels than the CO and EXE groups (Table 1).

**Table 1:** Anthropometry and nutritional and biochemical parameters of control animals (CO), animals submitted to exercise (EXE), cigarette smoke exposure (CS), and cigarette smoke exposure with exercise (CS+EXE).

	CO	EXE	CS	CS+EXE	P-value
Final weight (g)	459.40±44.21	435.80±35.21	434.00±63.50	437.10±34.12	0.553
Food consumption (g/day)	27.95±1.40^c^	27.80±1.6	26.03±1.76	28.49±1.43^c^	0.008
Ingested energy (kcal/day)	81.07±4.07^c^	80.64±4.77^c^	75.48±5.11	82.63±4.14^c^	0.008
Feed efficiency (%)	77.48±28.70	86.18±20.70	71.68±28.22	52.43±19.90^b^	0.030
Weight gain (g)	98.30±23.58	92.30±27.15	78.42±11.19^a^	69.30±27.47^a^	0.049
Body mass index (g/cm^2^)	0.73±0.04	0.68±0.02^a^	0.68±0.04^a^	0.72±0.05	0.048
Weight of prostate (g)	0.670±0.08	0.647±0.07	0.581±0.09	0.726±0.09	0.140
Relative weight of prostate	0.158±0.03	0.157±0.01	0.137±0.01	0.174±0.01	0.190
Triglycerides (mg/dL)	82.46±9.46^b^	111.85±23.92	83.41±9.92^b^	94.20±11.14	0.009
Cholesterol (mg/dL)	68.24±5.23^c.d^	72.84±5.61^c^	87.95±13.31	83.15±17.22	0.028
Glucose (mg/dL)	111.52±9.75	91.33±14.60	103.07±18.15	100.76±17.81	0.193

Data are reported as means±SD. ^a^P<0.05 *vs* CO; ^b^P<0.05 *vs* EXE; ^c^P<0.05 *vs* CS; ^d^P<0.05 *vs* CS+EXE, one-way ANOVA with Tukey's multiple comparisons *post hoc* test.

### Histological changes

**Figure 2:**
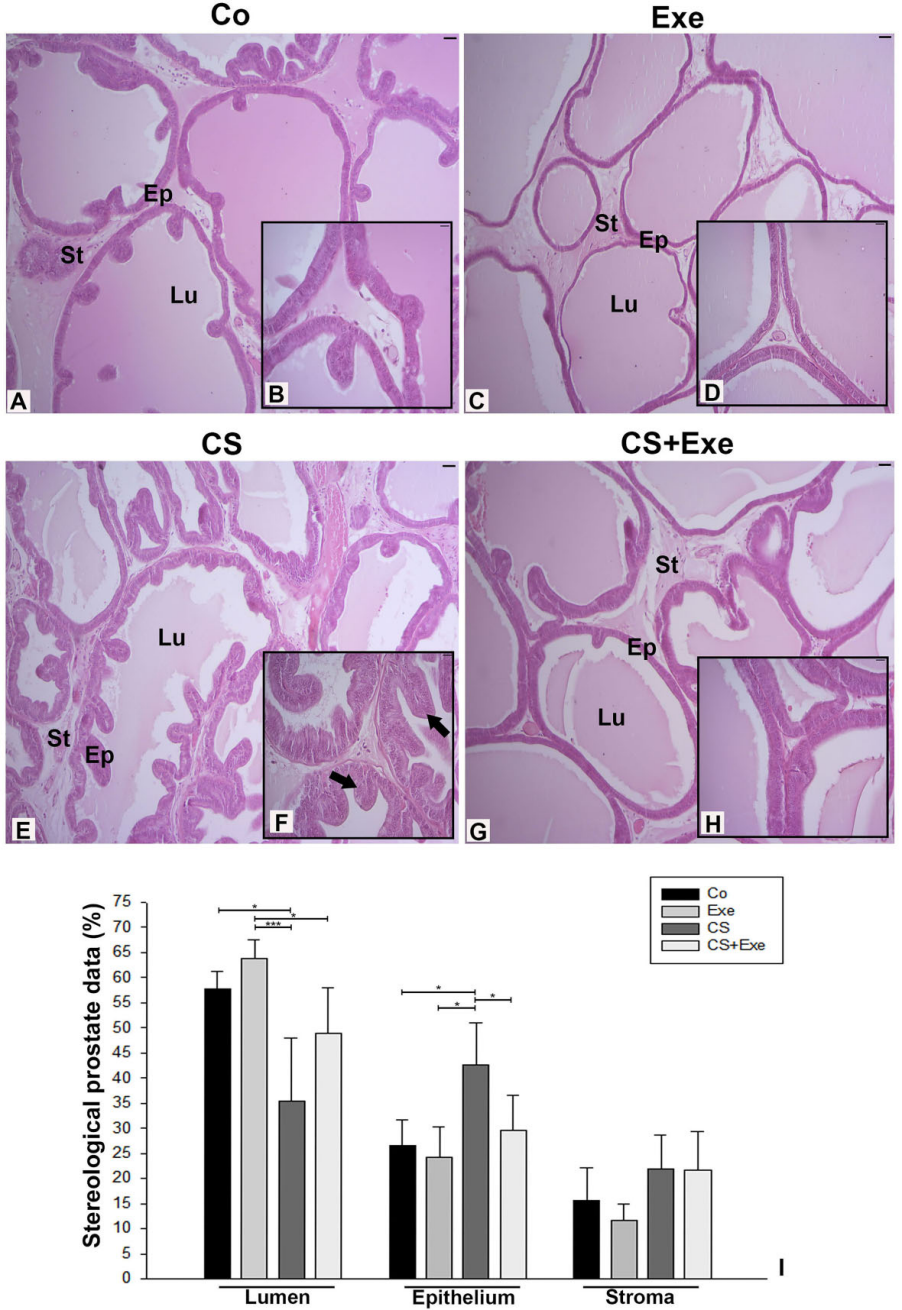
Histological analysis and quantification of stereology in the ventral prostate as a percentage of total prostate surface area from Wistar rats in the control group (Co) (n=10, **A** and **B**), exercise only group (Exe) (n=10, **C** and **D**), smoke exposure only group (CS) (n=8, **E** and **F**), and exercise with smoke exposure group (CS+Exe) (n=10, **G** and **H**). Images were captured at 10× magnification (scale bars, 50 µm) and enlarged to 40× magnification (scale bars, 20 µm) in the insets. Arrows indicate locations with prostatic intraepithelial neoplasia. Data are reported as mean percentage of surface area±SD. The Weibel reticle of 160 points was used for morphometric analysis and the stereology (acini, epithelium, and stroma) of prostate cross-sections were quantified across 10 images per slide, with 10 slides analyzed per prostate collected. *P<0.05, ***P<0.001, one-way ANOVA supplemented with Tukey multiple comparisons *post hoc* test. St: Stroma; Ep: Epithelium; Lu: Lumen.

To determine if cigarette smoke exposure and exercise, alone or in combination, altered the histological profile of the prostate, we examined prostatic epithelial volume and stroma as well as related stereology and proliferative protein expression. Secretory epithelium in the prostate with columnar and basal cells and other cells in the glandular stroma had a surface area of 26.6% epithelium, 57.6% acinar lumen, and 15.6% stroma in the CO group (Figure 2A, B, I). In the EXE group, the epithelium surface area was reduced to 24.3%, and the proportion of acinar lumen increased to 63.9%, though these changes were not significantly different from the CO condition (Figure 2C, D, I). Prostate columnar epithelium surface area in the CS group presented intraepithelial neoplasia as the proportion of epithelium was increased to 42%, significantly greater than all other conditions (Figure 2E, F, I, P=0.03). Interestingly, the exercise paradigm appeared to mitigate the increased prostatic epithelium caused by cigarette smoke exposure. In addition to epithelium changes, lumen decreases were also observed following smoke exposure in the CS (35.5%) and CS+EXE (32.7%) conditions (P=0.04). Although no significant lumen differences were observed between the CS and CS+EXE groups, the higher lumen content in the CS+EXE condition that was more reminiscent of the CO group may suggest a protective effect of exercise against smoke-induced prostate changes. However, despite no significant differences in stroma content across all conditions, both the CS and CS+EXE groups demonstrated a higher average stromal surface area than the CO and EXE groups (Figure 2I).

### Effects of cigarette smoke exposure and aerobic exercise on steroid receptors and apoptotic proteins

**Figure 3:**
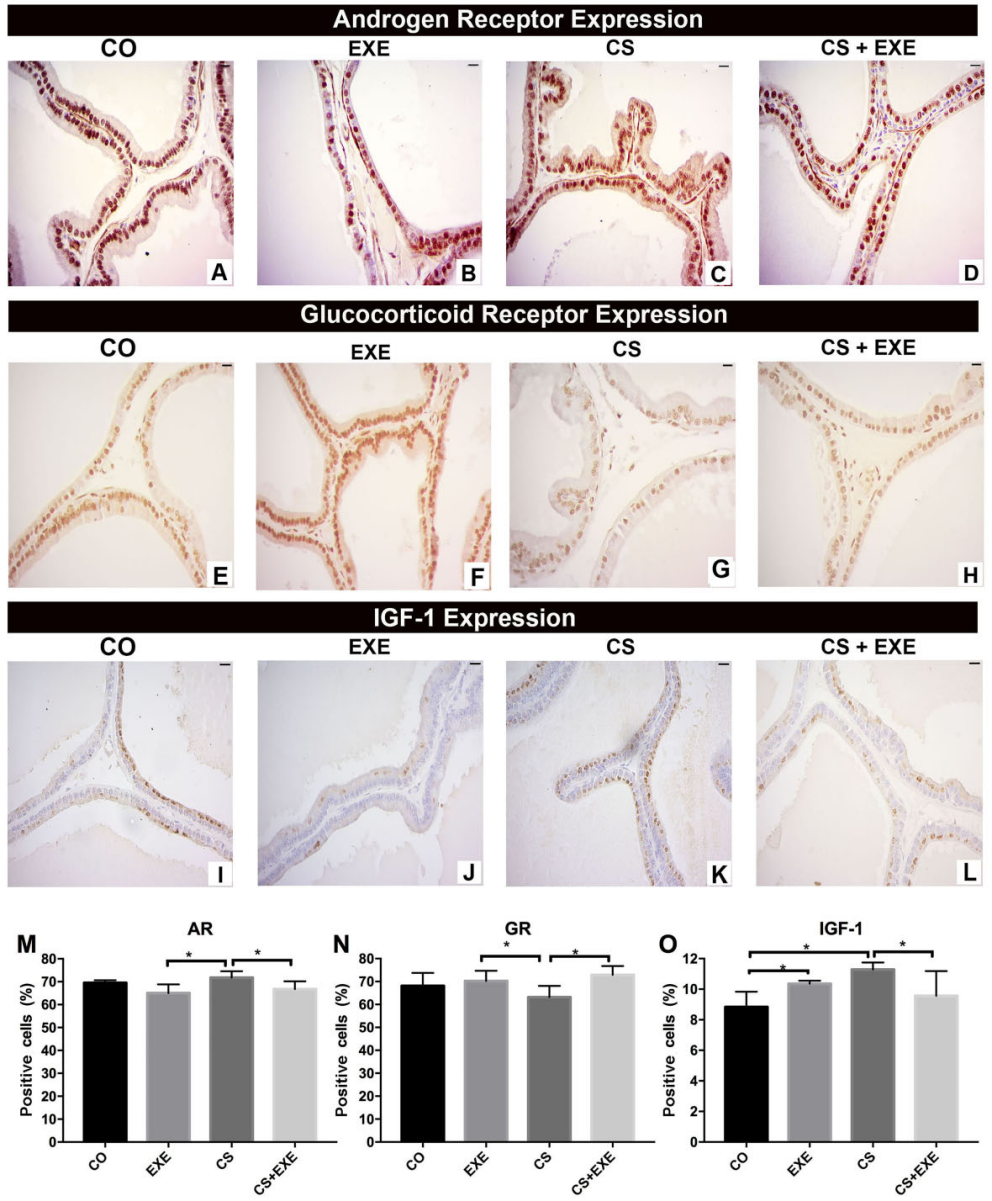
Distribution of the androgen receptor (AR), glucocorticoid receptors (GR), and insulin-like growth factor (IGF-1) expression evaluated by immunohistochemistry in the ventral prostate of Wistar rats submitted to cigarette smoke inhalation and aerobic exercise [control (CO) n=10; cigarette smoke inhalation (CS) n=8; exercise (EXE) n=10; cigarette smoke inhalation+exercise (CS+EXE) n=10]. AR expression is represented in images **A** to **D** and quantification of AR-positive prostate cells is shown in graph **M**. Representative images of GR expression are shown in images **E** to **H** and GR-positive cells are quantified in graph **N**. Images **I** to **L** are representative of IGF-1 expression, and the percentage of IGF-1-positive cells is shown in graph **O**. Scale bars, 20 µm. Data are reported as mean percentage±SD. *P<0.05, one-way ANOVA with Tukey's multiple comparisons *post hoc* test.

**Figure 4:**
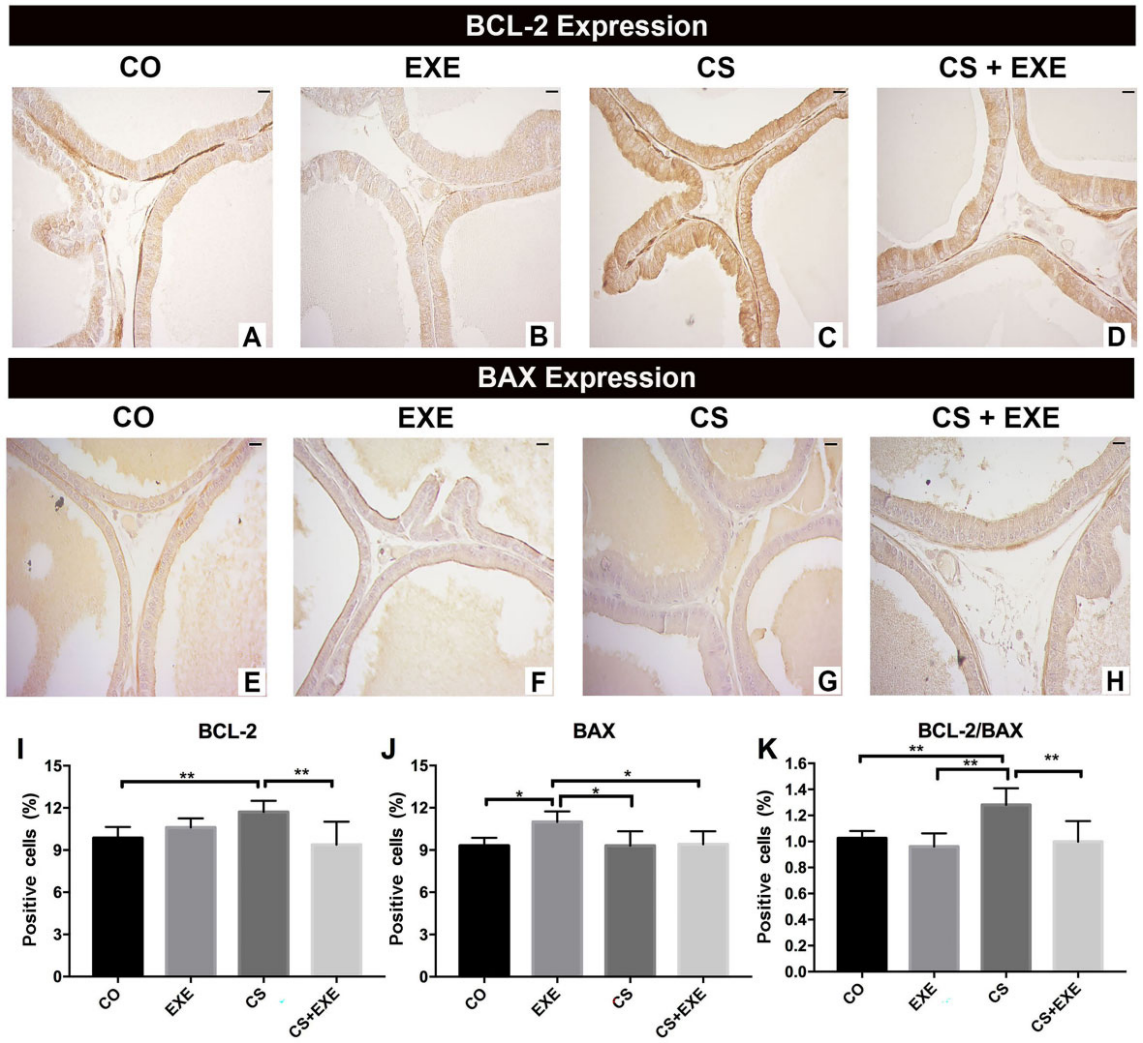
Distribution of BCL-2 and BAX protein expression evaluated through immunohistochemistry of the ventral prostate in Wistar rats undergoing inhalation of cigarette smoke and aerobic physical training [control (CO) n=10; cigarette smoke inhalation (CS) n=8; exercise (EXE) n=10; cigarette smoke inhalation+exercise (CS+EXE) n=10]. Representative images of BCL-2 and BAX expression are presented in **A** to **D** and **E** to **H**, respectively. Scale bars, 20 µm. Graphs **I** and **J** show the quantification of positive prostate cells for BCL-2 and BAX, respectively. The ratio of BCL-2/BAX expression in the prostate was quantified and is presented in **K**. Data are reported as mean percentage of protein-positive tissue surface area±SD. *P<0.05; **P<0.01, one-way ANOVA with Tukey's multiple comparisons *post hoc* test.

In the CS group, AR and IGF-1 protein expression were significantly higher than in the other groups, whereas AR expression was lowest in the EXE group; IGF-1 expression was lowest in the CS+EXE and EXE conditions (Figure 3A to D, M; I-L, O). Representative images of GR expression in the prostate of the CS group exemplify the low GR content in prostatic epithelium relative to all other conditions (Figure 3E to H, N). In contrast, GR expression in the EXE and CS+EXE conditions were comparable to observations in the CO condition, further suggesting a protective effect of exercise in prostate receptor expression. Similar to observations of AR and IGF-1 expression, levels of the anti-apoptotic protein BCL-2 and the ratio of BCL-2/BAX, a marker of prostate tumor proliferative potential, were higher in animals submitted to the CS protocol than in all other groups (Figure 4A to D, I, K). In contrast, BAX expression was highest in the EXE group relative to all other conditions (Figure 4E to H, J). This suggested that smoke exposure increased anti-apoptotic protein expression, dysregulated the ratio of BCL-2/BAX, and increased AR and IGF-1, inducers of cell proliferation. Exercise mitigated these changes. Furthermore, a positive correlation between epithelium volume and BCL-2 expression was observed under cigarette exposure conditions (r=0.447, P=0.048).

### Physical exercise reduced prostatic proinflammatory cytokines

**Figure 5:**
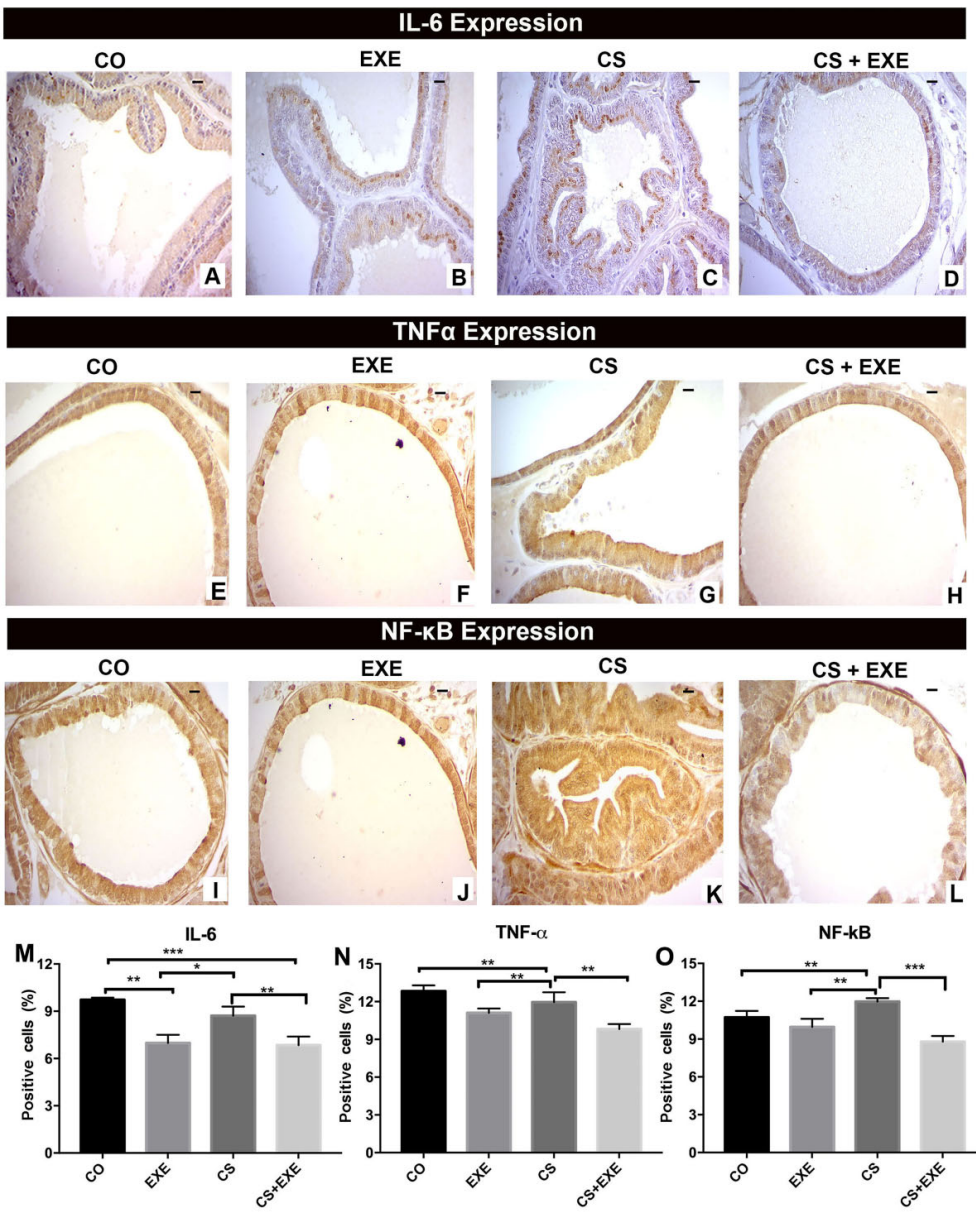
Distribution of interleukin (IL)-6, tumor necrosis factor-alpha (TNF-α), and nuclear factor-kappa B (NF-κB) protein in the ventral prostate of Wistar rats submitted to cigarette smoke inhalation and exercise as evaluated using immunohistochemistry [control (CO) n=10; cigarette smoke inhalation (CS) n=8; exercise (EXE) n=10; cigarette smoke inhalation+exercise (CS+EXE) n=10]. Representative images of IL-6, TNF-α, and NF-κB are shown in images **A** to **D**, **E** to **H**, and **I** to **L**, respectively. Scale bars, 20 µm. Graphs **M**, **N**, and **O** show the quantification of positive prostate cells for IL-6, TNF-α, and NF-κB, respectively. Data are reported as mean percentages of protein-positive tissue surface area±SD. *P<0.05; **P<0.01, ***P<0.001, one-way ANOVA with Tukey's multiple comparisons *post hoc* test.

Representative images of prostatic IL-6, TNF- α, and NF-κB expression in each group are presented in Figure 5A to L. The EXE and CS+EXE protocols reduced the expression of pro-inflammatory IL-6 compared with expression in the CO and CS groups (Figure 5M). Additionally, the EXE and CS+EXE groups also showed reduced TNF-α expression compared with the CS group (Figure 5N). In contrast, the CS group presented the highest NF-κB expression compared with all other groups (Figure 5O). These results suggested smoke exposure promoted inflammation and decreased GR expression. However, aerobic physical training in both the CS+EXE and EXE groups reduced the expression of NF-κB, TNF-α, and IL-6, and increased GR expression.

## Discussion

**Figure 6:**
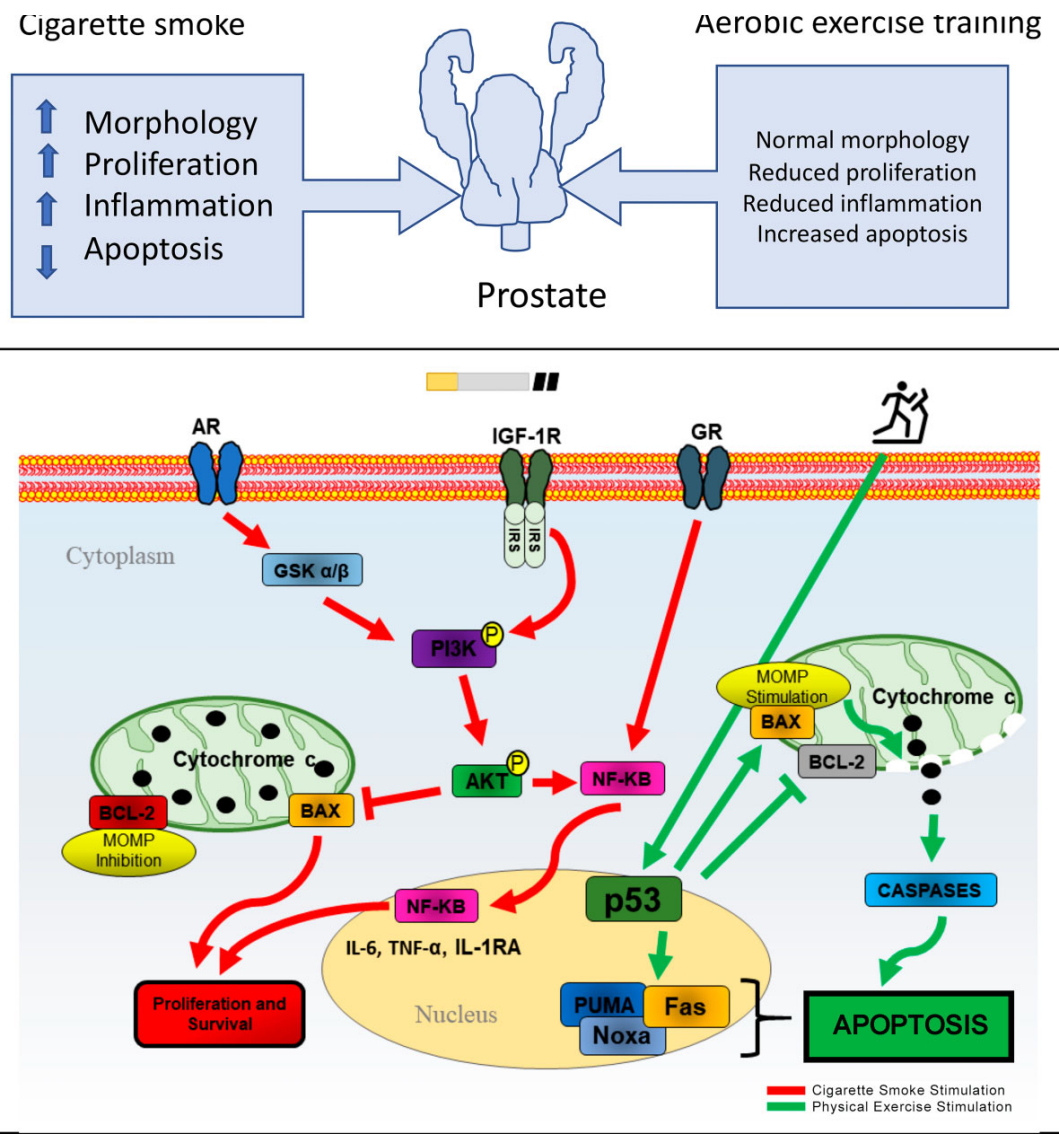
Central role of cigarette smoking on prostate inflammation in the progression of dysregulation and the effects of physical exercise. The prostate is a steroid- and immune-competent organ characterized by complex molecular pathways considered to be triggers for the dysregulation of the prostatic immune system and the development of chronic prostatic inflammation. Cigarette smoking promotes hormonal receptor and growth factor activation, which can trigger the phosphorylation of the PI3K and AKT pathways, inhibiting BAX and stimulating nuclear factor-kappa B (NF-κB) activity. AKT prevents cytochrome C release and inhibits apoptosis following cigarette smoking. Regular physical exercise can regulate the activation of the PI3K/AKF/NF-κB pathway and promote glucocorticoid receptors (GR) activation by reducing NF-κB and cytokine expression, possibly by p53 activation and subsequent BAX upregulation, which leads to apoptosis and proliferative reduction. AR: androgen receptor; MOMP: mitochondrial outer membrane permeabilization.

Our findings demonstrated that: a) cigarette smoke reduced food consumption, weight gain, and BMI values, and regulated food consumption and body weight; b) exercise increased blood triglyceride concentrations and decreased cholesterol and glucose, although smoke exposure increased cholesterol; c) smoke exposure upregulated AR and IGF-1 expression and reduced GR with concomitant increases in anti-apoptotic protein BCL-2 and prostate epithelium volume; d) pro-apoptotic protein BAX was upregulated following exercise and downregulated after smoke exposure; and e) exercise reduced inflammatory cytokines in the prostate, and smoke exposure increased the expression of NF-κB. Thus, these results suggested that cigarette smoke inhalation promoted the expression of proliferative, anti-apoptotic, and inflammatory compounds in the prostate. These changes may be reversed with exercise, as aerobic physical training increased the expression of pro-apoptotic proteins and reduced inflammation, maintaining homeostasis in prostatic tissue. The proposed relationships between physical exercise, cigarette smoke exposure, prostatic health, and apoptotic and proliferative balance are summarized in Figure 6.

We observed changes suggesting that cigarette smoke exposure reduced weight gain by modulating feed efficiency, and consequently weight gain. The regulation of appetite and ingested energy are fundamental for maintaining caloric balance and body weight, and previous studies have also shown that administering cigarette smoke to rats reduces body weight (15). The anorexic effects of tobacco are attributed to nicotine (11). In contrast, aerobic physical exercise reduces BMI and improves feed efficiency in rats. However, cigarette smoke inhalation in combination with physical exercise reduces weight gain and feed efficiency, compared to exercise or smoke inhalation alone, even following high caloric intake. Both cigarette smoke and aerobic training increase metabolic rates and thermogenesis in adipose tissue, stimulate lipolysis, and reduce food intake, and may act on the recycling of fatty acids in triglycerides, thus contributing to lower body fat indexes (16). Nevertheless, the literature links cigarette smoking with visceral fat increase, enhanced cholesterol levels, greater waist-to-hip ratio, and atherosclerotic cardiovascular disease, and suggests that reduction in body weight by smoking is non-beneficial to health (17).

Smoking may promote dyslipidemia (18) as observed in this study showing that smoke inhalation increases cholesterol and glucose levels. In support of this, many studies have shown an association between dyslipidemia and benign prostatic hyperplasia (19). Mounting evidence suggests that metabolic syndrome is correlated with smoking (16). The relationship between smoking, dyslipidemia, and associated metabolic syndrome could play a role in the development and progression of prostate cancer (20). Mostaghel et al. (21) found that each 10 mg/dL increase in total cholesterol above the abnormal cut-off value of 200 mg/dL was associated with a 9% increased risk of prostate cancer. Our results demonstrated that cigarette smoke exposure increased cholesterol, whereas aerobic physical training reduced cholesterol. Morales-Palomo and colleagues (22) found that aerobic exercise reduces metabolic syndrome by improving cardiorespiratory function. Thus, the effectiveness of physical training on reducing cholesterol levels reported in this study corroborated current literature findings (23). Aerobic exercise possibly recovered smoke-induced consequences on prostate health by reducing the effects associated with metabolic syndrome. The groups exposed to cigarette smoke showed a positive correlation between total cholesterol levels with prostatic epithelium (r=0.554, P=0.01) and BCL-2 expression (r=0.447, P=0.048). In summary, we found strong evidence to support the link between total cholesterol levels in serum and the risk of cell proliferation in the prostate.

Interestingly, aerobic physical training downregulated the expression of AR in prostate cells, which was associated with reduced proliferation in the prostatic epithelium. Cigarette smoke inhalation, in contrast, was associated with significantly higher AR, IGF-1, and BCL-2 expression in the prostate. High prostatic AR expression induced by cigarette smoke inhalation is associated with elevated levels of testosterone and decreased estradiol (24). Stone and colleagues (19) previously found that smokers with prostate cancer show higher than normal levels of plasmatic testosterone, which is associated with CYP3A expression, a proliferative inducer. Likewise, our findings showed that cigarette smoke exposure promoted proliferation in the prostate, potentially stimulated by AR and IGF-1 activity. Increased testosterone-induced AR expression may increase expression of the IGF-1 receptor (25); elevated IGF-1 expression activates the phosphorylation of subsequent proteins in the PI3K/AKT pathway (26)(32). Phosphorylation of AKT by AR and IGF-1 activity stimulates BCL-2 and BCL-XL, inhibiting the release of cytochrome C from mitochondria and preventing cell apoptosis while promoting cell proliferation (26). These findings are further substantiated by our observations that prostatic proliferation was greater in animals following cigarette smoke inhalation than those that did not inhale smoke. Furthermore, physical exercise reduced activation of AR and IGF-1 in the prostate, possibly by reducing the expression of free testosterone (27).

Cigarette smoke, which induces oxidative stress, protease release, and drives inflammatory response, is considered a factor in the development of prostatic diseases (28). Previous findings show that GR expression is significantly lower in animals exposed to cigarette smoke, leading to increased transcription of pro-inflammatory genes (7). Considering that GR plays an integral role in the regulation of inflammatory responses in the prostate, we further investigated GR expression in response to cigarette smoke exposure and found reduced GR expression. GR may act via protein-protein interactions with other transcription factors such as activator protein-1, Smad3, and NF-κB (29) to indirectly alter gene transcription. Our previous work demonstrates that the anti-inflammatory effects of GR activity occur through a DNA-independent mechanism (5). Glucocorticoids have been shown to block NF-κB activation by multiple mechanisms in several cell types (7), including binding of GR with NF-κB to suppress NF-κB-induced inflammatory gene expression in the prostate. Our present results demonstrated that smoke inhalation reduced GR expression and increased NF-κB, despite no differences in IL-6 and TNF-α expression. The suppression of GR activity by p65 requires phosphorylation at S276 by protein kinase A and GR-mediated inhibition of NF-κB activity is also protein kinase A-dependent. Thus, the cross-suppression of NF-κB and GR activity is regulated by protein kinase A-associated signaling (30). However, physical exercise restores GR protein expression, which might subsequently attenuate smoking-induced inflammatory response and improve NF-κB and IL-6 activity in the prostate.

In the present study, we observed that chronic exposure to cigarette smoke-induced characteristics consistent with prostate cancer pathology and intraepithelial neoplasm, including increased epithelium volume, inflammation, and a proliferative state. Additionally, we found that aerobic physical exercise decreased the inflammatory proteins IL-6, TNF-α, and NF-κB in prostate tissue. Petersen and Pedersen (31) suggested that during an inflammatory process, cytokines produced by adipose tissue such as TNF-α are recruited and stimulate the production of IL-6 and the interleukin receptor antagonist-1. When produced and mediated during exercise, IL-6 inhibits the effects of TNF-α and enhances expression of anti-inflammatory cytokines such as IL-10, inhibiting the production of inflammatory proteins such as IL-1β, TNF-α, and IL-1α.

Collectively, these results suggest that physical exercise is effective in protecting prostate function and suppressing the inflammatory response. Thus, we have demonstrated that physical exercise may promote apoptosis in prostatic cells by inhibiting androgen stimulation and the action of growth factors (5), further supported by alterations in BCL-2/BAX ratios seen here that suggested increased apoptosis. It has previously been established that BAX is a potent apoptosis inducer through the action of its target genes, such as p53. While we do not know the exact molecular mechanisms underlying apoptotic modulation in aerobic physical exercise, we hypothesized that exercise may stimulate p53 phosphorylation, increasing phosphorylated JNK and p38 MAPK, nuclear c-JUN, and activator protein-1 DNA-binding activity. In support of this, Camera et al. (32) found that physical exercise increases the expression of p38MAPK, p53, and PGC-1-α. We observed that aerobic physical training directly or indirectly regulated apoptosis in the prostate as reflected by decreased BCL-2 levels compared to the cigarette smoke exposure-only group. These data suggest that physical exercise may protect against the development of tobacco-induced prostate diseases.

There are some notable limitations to this study. Firstly, the animals in our study were exposed to an 8-week regimen of smoke exposure, and it is possible that a longer exposure period could produce more extensive changes to the prostate that would better recapitulate prostate morphology changes observed in clinical populations. Secondly, the molecular pathways in the prostate associated with aerobic physical exercise have yet to be identified, and this study did not explicitly evaluate the mechanisms associated with smoke- and exercise-related changes to prostate health. Additionally, as previously discussed, cigarette smoke exposure can induce oxidative stress in the prostate, which may impact the epithelium. However, our study did not evaluate the role of oxidative stress and p53 function associated with physical exercise. Future research should be conducted to increase understanding of how physical exercise and smoke exposure modulate prostate health at the molecular level, and how variables other than those investigated in this work could affect prostate health in the context of smoking and exercise.

In conclusion, our results demonstrated that cigarette smoke exposure altered biochemical parameters and exacerbated cell proliferation in the prostate. In contrast, aerobic exercise improved body and prostate weight, decreased AR and IGF-1, and regulated the BLC-2/BAX index towards increased apoptosis. These observations indicated that chronic exposure to cigarette smoke may anticipate the onset of prostatic diseases caused by increased cell proliferation, although regular exercise may maintain prostate tissue homeostasis. The expected outcomes of anti-tumor therapies are increased apoptosis and reduced cell proliferation. Physical exercise has demonstrated effective apoptotic action and, as seen in this study, anti-inflammatory effects in the prostate. Further research is needed to delineate the mechanisms mediated by aerobic physical exercise in the prostate and to determine optimal intensity, duration, and type of exercise to optimize the beneficial effects on cellular health and the prostate while minimizing the adverse effects of smoke exposure.
